# Extreme lower jaw elongation in a placoderm reflects high disparity and modularity in early vertebrate evolution

**DOI:** 10.1098/rsos.231747

**Published:** 2024-01-31

**Authors:** Melina Jobbins, Martin Rücklin, Marcelo R. Sánchez Villagra, Hervé Lelièvre, Eileen Grogan, Piotr Szrek, Christian Klug

**Affiliations:** ^1^ Department of Palaeontology, University of Zurich, Karl-Schmid-Strasse 4, 8004 Zurich, Switzerland; ^2^ Naturalis Biodiversity Center, Darwinweg 2, 2333 Leiden, The Netherlands; ^3^ University of Leiden, Sylviusweg 72, 2333 Leiden, The Netherlands; ^4^ 101 Muséum National d'Histoire Naturelle, 57 rue Cuvier, 75005 Paris, France; ^5^ Department of Biology, Saint Joseph's University, 5600 City Avenue, 19131 Pennsylvania, PA, USA; ^6^ Polish Geological Institute–National Research Institute, Rakowiecka 4, 00-975 Warsaw, Poland

**Keywords:** placoderm, gnathostome, modularity, disparity, jaw elongation

## Abstract

Jaws are a key vertebrate feature that arose early in our evolution. Placoderms are among the first jawed vertebrates; their fossils yield essential knowledge about the early diversification of gnathostome feeding strategies, diets and modularity. Modularity can be expressed through disproportional lengths of lower and upper jaws as in swordfish or halfbeaks. *Alienacanthus malkowskii* is an arthrodire from the Famennian of Morocco and Poland, whose most remarkable feature is its lower jaw, which is twice as long as the skull. This is the oldest record of such extreme jaw elongation and modularity in vertebrates. The gnathal plates of *Alienacanthus* possess sharp, posteriorly recurved teeth that continue anterior of the occlusion in the inferognathals. The dentition suggests a catching and trapping live prey function, and the jaw occlusion is unique among placoderms. This armoured ‘fish’ expands the morphological and ecological diversity during one of the first radiations of jawed vertebrates with a combination of features so far unrecorded for arthrodires.

## 1. Introduction

Placoderms are the phylogenetically earliest jawed vertebrates [1]. They play an important role for the understanding of the origin, early evolution and diversification of structures such as jaws and teeth. In the Devonian, placoderms have the highest diversity among jawed fishes and probably occupied many different habitats [2,3]. In particular, arthrodires are the most common and diverse placoderm group. They display a high disparity through time, providing a record of the functional and ecological variation of an early jawed vertebrate evolutionary radiation [4–6]. For example, the recent discovery of a selenosteid preserving its body outline [7] provided new insights into its ecology and swimming abilities. However, body contours and stomach contents are rarely preserved in arthrodires [7–10] and, among the typically preserved armoured plates of the group, gnathal (jaw) elements yield the most information regarding ecological diversity, including feeding. Early placoderms show little jaw variation compared to later forms as jaws from the earliest taxa were adapted to fast closure and stress resistance [10]. The Late Devonian presents a much higher diversification of jaw morphology, most of which favoured a variety of feeding strategies over fast closure [11,12], including filter feeding [13] and durophagous diets [14–16].

The fossil presented here is that of an arthrodire first described as a putative placoderm [17,18] from the Late Devonian of Poland. Two nearly complete skulls with jaws were discovered in the eastern Anti-Atlas of Morocco in a similar stratigraphic position, enabling us to better describe the species and to evaluate the phylogenetic position of *Alienacanthus*. We discuss jaw modularity and possibilities regarding functional morphology and feeding ecology of this taxon. We compare the jaw articulation and structures of the hyoid apparatus to that of other early vertebrates to improve our understanding of their use in this animal with unusual jaw morphology.

## 2. Material and methods

### 2.1. Localities and storage

**Figure 1.**
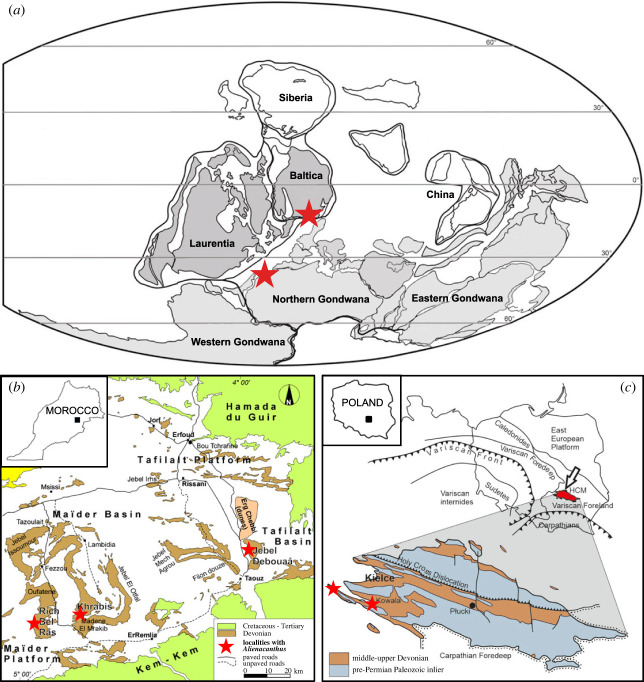
Map of the Late Devonian (Famennian) showing the *Alienacanthus* localities.

The specimens described here (electronic supplementary material, table S1) were found in the Moroccan Anti-Atlas and the Polish Holy Cross Mountains (figure 1). The Moroccan specimens are stored at the Université Cadi Ayyad, Faculté des sciences et techniques, Département des sciences de la terre, Laboratoire Géosciences et Environnement in Marrakech, Morocco (AA.MEM.DS), the Muséum National d'Histoire Naturelle in Paris, France (MCD), and the Department of Palaeontology of the University of Zurich, Switzerland (PIMUZ). The Polish specimens are at the University of Warsaw, Museum of the Earth of the Polish Academy of Sciences, and Polish Geological Institute-National Research Institute, Warsaw, Poland (MWG UW, MZ VIII Vp and Muz. PGI-NRI). The Paris material comprises three specimens found in Jbel Debouâa, located in the southern part of the Tafilalt Basin (figure 1*b*). Conodonts found in the matrix were identified as *Palmatolepis perlobata helmsi*, *Scaphignathus velifer velifer*, *Melhina strigosa* and *M*. cf. *strigosa*, dating the fossils to the middle to late Famennian (*trachytera* conodont zone) [19,20]. The other Moroccan materials come from the localities of Rich bel Ras and Madene El Mrakib in the Maider basin, in (figure 1*b*) and date back to the middle to late Famennian *Platyclymenia annulata* ammonoid zone [21]. The Polish specimens were excavated in Ostrówka Quarry (Gałęzice) and Kowala Quarry, located in the western area of the Holy Cross Mountains (figure 1*c*). The fossils date back to the Clymenia beds of the *trachytera* conodont zone [17,18]. Fieldwork including fossil collecting in Morocco was authorized through permit no. 1571/DE/DG, provided by the Ministère de l'Energie, des Mines et de l'Environnement.

### 2.2. Preparation

The material stored in Paris was prepared by formic acid dilution and additional mechanical preparation was performed with needles. Intermediate steps in the preparation process were photographed to record the natural contacts between the dermal plates. The material stored in Zurich was mechanically prepared with a combination of air-scribes and sandblaster. Two small portions of the right inferognathal (IG) and the tip of the left one were reconstructed in the small lower jaws. Thin sections of gnathal and dermal shield elements were performed in the Muséum National d'Histoire Naturelle, Paris for their material and in the University of Warsaw for the Polish specimens.

### 2.3. Phylogenetic analysis

The character matrix is based on the 98 characters for 28 taxa in Jobbins *et al*. [7], to which six taxa obtained from Trinajstic & Dennis-Bryan [22] were added: *Pachyosteus*, *Panxiosteus*, *Janiosteus*, *Plourdosteus*, *Protitanichthys* and *Fallocosteus*. The character matrix (electronic supplementary material, table S2) was converted into a nexus file using Mesquite [23]. *Dicksonosteus*, *Holonema*, *Buchanosteus* and *Homosteus* were defined as outgroups, following Rücklin *et al*. [24], to determine character polarity. As in Jobbins *et al*. [7], characters 4, 14, 20, 35, 51, 75, 92 and 93 were ordered, as these were changing sequentially across a gradient of states. The analysis was performed using a parsimony heuristic search in PAUP [25] with the random addition sequence of 10 repetitions and the holding 100 trees-option. All trees were rooted using the **‘**make outgroup paraphyletic with respect to ingroup’ option. Description of the characters is provided in the electronic supplementary material, info. S2.

### 2.4. Abbreviations

MNHN, Muséum National d'Histoire Naturelle, Paris, France; PIMUZ, Palaeontological Institute and Museum, University of Zurich, Switzerland; MZ, Museum of Earth, Polish Academy of Sciences, Warsaw, Poland; MWG UW, Faculty of Geology, University of Warsaw, Warsaw, Poland, Muz. PGI-NRI, Polish Geological Institute-National Research Institute.

## 3. Results

### 3.1. Systematic palaeontology

PLACODERMI McCoy, 1875

ARTHRODIRA Woodward, 1891

SELENOSTEIDAE Dean, 1901

*Alienacanthus* Kulczycki, 1957

**Type species**: *Alienacanthus malkowskii* Kulczycki 1957, [17, pl.13, fig. 1*a–c*].

**Figure 2.**
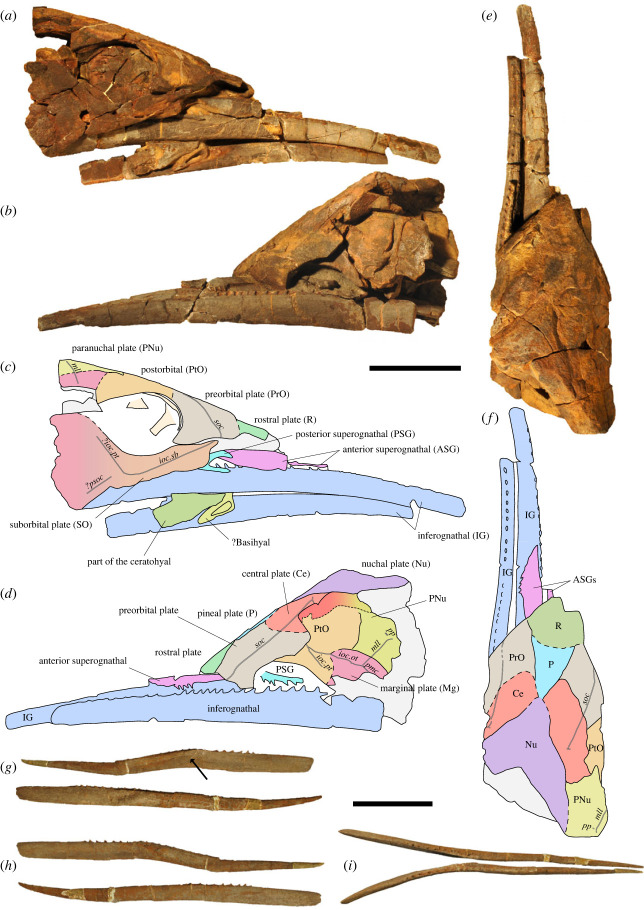
*Alienacanthus malkowskii*, skull, PIMUZ A/I 5239. In right (*a,b*), left (*c,d*) and dorsal (*e,f*) view; inferognathals, PIMUZ A/I 5238, in lingual (*g*), lateral (*h*) and dorsal (*i*) view. Each bone is differentiated by a separate colour. Black arrow points to lingual depression. Scale bars correspond to 100 mm.

**Remarks**. The preservation of several features is the basis for an emended diagnosis (see the electronic supplementary material, info. S1 for full description of the features), because the original diagnosis was based solely on isolated material, described as **‘**…extremely large, paired and unpaired spines, built of osseous tissue’ [17, p. 357]. In fact, the supposed **‘**spines’ are IGs [18]. The Moroccan material (figure 2; electronic supplementary material, figures S1 and S2) has the same age as the new Polish material, which is from the same locality and strata as the type material (electronic supplementary material, figures S3 and S4). The Moroccan and Polish IGs have the same shape. The holotype and the new specimens present an elongate and slender jaw with posteriorly recurved teeth. The curvature of the teeth is less pronounced in the holotype because they are worn, which is also seen in worn teeth at the anterior parts of the gnathal plates from Morocco. Recently discovered material from the type locality displays the same tooth curvature. The IG shape is similar in cross section throughout the plate in both the Polish and the Moroccan material, including the holotype. An inferognathal alone provides only little insight into the anatomy of the animal. Thus, we assign the new material to the type species and designate the two best-preserved specimens as epitypes for a better species referral.

**Holotype**: MZ VIII Vp-45, a 32 cm long fragmentary IG.

**Epitype 1**: PIMUZ A/I 5239, a nearly complete articulated skull with all gnathal elements.

**Epitype 2**: MCD 201, left side of partially preserved skull with all gnathal elements.

**Referred material**. MZ VIII Vp-44, -42, -47, fragmentary IGs; PIMUZ A/I 5238, a set of small paired IGs; AA.MEM.13, fragmentary IG; AA.MEM.14, sclerotic ring and skull elements; MCD 200, fragmentary right IG; MCD208, incomplete left IG; MCD 209, posterior lamina of right IG; MCD 210, isolated right anterior superognathal (ASG). MWG UW ZI/43/0077, left partial IG; MWG UW ZI/109/1, putative postorbital; Muz. PGI-NRI 1809.II.24 and 1809.II.25, fragmentary IGs; 1809.II.35, incomplete IGs on matrix.

**Age**: *trachytera* conodont zone, middle Famennian, Late Devonian.

**Localities.** Morocco: Jbel Debouâa, southern Tafilalt; Rich bel Ras and Madene El Mrakib, Maider Basin. Poland: Ostrówka quarry, near Gałęzice, Kowala Quarry, western Holy Cross Mountains.

**Emended diagnosis**. Large eubrachythoracid with long protruding IGs that bear a series of in-line teeth on the labial edge of the jaw, which are posteriorly recurved. The IGs reach about twice the length of the skull including the superognathals and lack traces of attachment for the mentomeckelian cartilage. Both superognathals have a vertical dorsal process instead of horizontal and have sharp recurved teeth posteriorly and worn teeth anteriorly. The ASGs protrude shortly anterior of the rostrum. The dermal plates of the skull-roof show no ornamentation. The rostral plate is pronounced, deprived of the laterally developed wings anteriorly, and its simple non-jagged contact with the pineal plate separate the preorbitals. The preorbital and central plates are of subequal length. The central plates only join at a single contact point with the pineal and nuchal plates. The orbits are large, and the suborbital plates possess a well-developed linguiform process. The embayment of the nuchal plate is deep and therefore the nuchal gap is probably large.

### 3.2. Description

#### 3.2.1. Skull

The epitypes PIMUZ A/I 5239 (figure 2*a–c*) and MCD 201 (electronic supplementary material, figures S1 and S2) provide data about most cranial elements, including the paired preorbital, postorbital, suborbital, central, marginal and paranuchal plates, the single rostral, pineal and nuchal plates, gnathal plates, parasphenoid and hyoid elements (detailed description in the electronic supplementary material, info. S1). The cranium is funnel-shaped with a rounded cross section throughout. Measurements of each specimen are provided in table 1. The orbits are large and drop-shaped with the tip pointing postero-dorsally. The proportions of the orbits are reminiscent of those of selenosteids, a derived group of pachyosteomorphs with large eyes.

**Table 1.** Measurements and sizes of main *Alienacanthus* specimens. (Estimations are marked with an asterisk (*) and incomplete in red. Values are in mm. (l) is for left element and (r) is for right element. IG, inferognathal; ASG, anterior superognathal; PSG, posterior superognathal.)

	PIMUZ A/I 5239	PIMUZ A/I 5238	MCD 201	MCD 200	MCD 210
length of specimen	420	—	460	—	92
length of skull (incl. IG)	500*	—	800*	—	—
length of skull (without IG)	250*	—	370*	—	—
length of inferognathals	500*	410	800*	340	—
orbit length					
(anterior to posterior)	100	—	—	—	—
number of teeth ASG	7(l)	—	4	—	6*
number of teeth PSG	5(l)	—	10	—	—
number of teeth IG	25(l) 24(r)	13(l) 18(r)	21(l) 8(r)	12	—

#### 3.2.2. Skull plates

The rostral and pineal plates separate the preorbitals (PIMUZ A/I 5239; figure 2). There is a single, quadruple contact point between the pineal, both central, and nuchal plates. The nuchal plate is large, and its posterior margin is strongly concave, implying a large nuchal gap as in the selenosteids *Draconichthys* [26], *Microsteus* [27] or *Gymnotrachelus* [28]. A large nuchal gap implies the presence of well-developed nuchal muscles involved in movements raising the head [29,30]. The orbital margin is formed by the preorbital, postorbital, suborbital and marginal plates. The paranuchal plates are rather small and have a short posterior pitline groove near the posterior edge. The suborbital plates are slender, and have a well-developed linguiform process, which articulates with the autopalatine.

#### 3.2.3. Gnathal plates

The Moroccan specimens preserve all three paired elements comprising the jaws: the IG (lower jaw), and the anterior and posterior superognathals (PSGs; upper jaws). The IGs are prognathous; they are about twice as long as the skull. The occlusal surface extends a little anteriorly to the rostrum. The upper elements (superognathals) are straight and flattened, and the dorsal processes are on a similar dorsoventral axis as the rest of the elements, a unique feature among placoderms (electronic supplementary material, figure S5). All gnathal plates bear posteriorly recurved teeth. The posterior ones are slender and sharp, with very little gradual wear, while the anterior ones are abruptly worn. The ASG is a solid laterally flattened plate with a thin occlusal margin and dorsal process forming a laterally acute angle with the occlusal margin. The tip of the ASG is pointed, as is that of the IGs. The PSG is slender, laterally flattened, and has a short and dorsally developed dorsal process. The IGs are the best preserved and most commonly found elements of *Alienacanthus*. The IG is a long and slender bone with a sharp anterior tip, Y-shape in dorsoventral view, and tooth row extending past the occlusal region. In arthrodires, the IG usually comprises a biting division (anterior) and a blade, or internal shaft (posterior). *Alienacanthus* presents an additional primarily toothless section anterior to the biting division. The right and left elements were anteriorly in close contact over *ca* 60% of the jaw length, presenting a general jaw morphology reminiscent to other long jawed animals like mixosaur ichthyosaurs [31] or Hemiramphidae (halfbeaks) [32]. A depression on the medial side of the IGs occurs where the elements meet posteriorly (figure 2*g*). The smooth tip of the IG of PIMUZ A/I 5238 does not show traces of contact with the mentomeckelian cartilage.

#### 3.2.4. Histology of the gnathal plates

**Figure 3.**
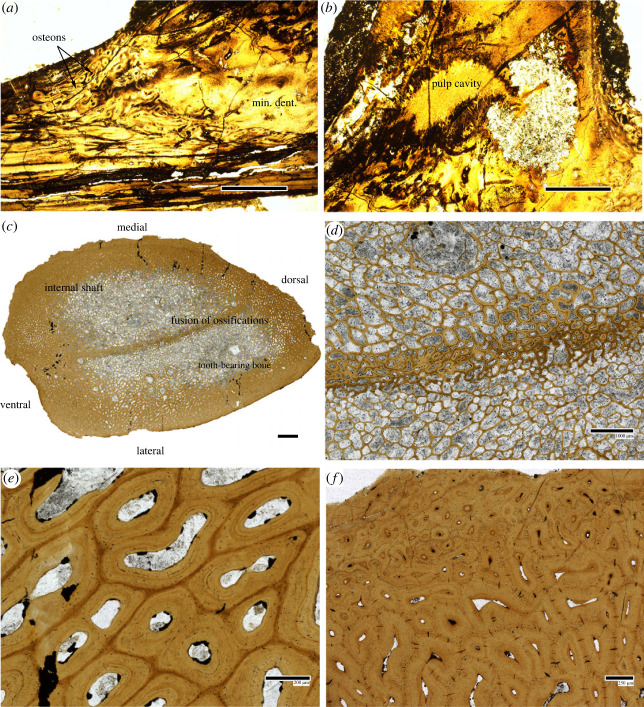
Histology of *Alienacanthus*. Section of an ankylosed tooth from MCD200 (*a,b*) and inferognathal from 1809.II.24 (*c–f*), overview (*c*), detail of the area where both ossifications meet (*d*), dense outer layer (*e,f*). Tiny bone cell lacunae and multiple cracks are respectively visible in (*e*) and (*f*). Scale bars correspond to 1 mm (*a,b,d*), 2 mm (*c*), 200 microns (*e*), and 250 microns (*f*).

Two ossifications are discerned in the IG (figure 3). The first holds the teeth (biting division) and lies laterally, with slight elevation, next to the second (internal shaft). Both ossifications have a dense, poorly vascularized, outer layer surrounding a well-vascularized trabecular bone (figure 3*c*). The bones meet centrally to form a fused margin but there is no clear separation between both ossifications (figure 3*d*). Bone cell lacunae are present in the interstices between the osteons of the dense bone layer, particularly in the medial part (figure 3*e*). The cell lacunae are rounded and connected through canaliculi. The superficial medial layer also yields cracks at the interstitial zone (figure 3*f*), especially in the least vascularized part of the layer; these reach into the first few outer osteon concentric lamellae and are perpendicular to the osteon margin. The teeth are ankylosed to the jaw and show strong recrystallization but yield dentinous tissue, most likely semidentine, and a pulp cavity (figure 3*a,b*). There is no presence of enamel.

#### 3.2.5. Hyoid elements

Both Moroccan skulls, PIMUZ A/I 5239 and MCD201, preserve structures interpreted as the first branchial arch of the hyoid apparatus (figure 2; electronic supplementary material, figure S2). A partially visible putative basihyal is located where both IGs meet in the first epitype, exhibiting a ventral deep pit. The ceratohyal lies next to the latter; it is triangular and surrounds the margins of the IG laterally and labially. An oblong segment branches from the posterior end of the triangular section of the ceratohyal in the second epitype.

### 3.3. Phylogeny and *Alienacanthus* affinities

**Figure 4.**
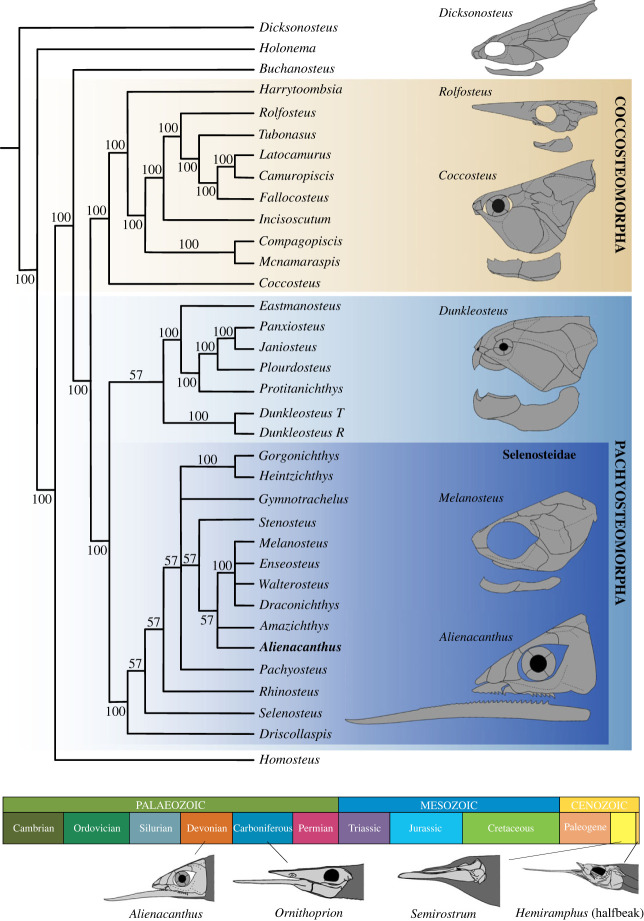
Eubrachythoracid phylogeny including *Alienacanthus* and simplified morphological diversity of skulls and jaws in different groups. Based on the computed 50% majority-rule consensus tree. Outlines of *Dicksonosteus*, *Rolfosteus*, *Coccosteus*, *Dunkleosteus*, and *Melanosteus* are modified from [8,33–35], and [36]. Comparison to other known taxa with an elongated lower jaw through time. Reconstructions not to scale and lower jaws are separated from the skull to show their shapes.

The parsimony analysis of the character matrix (see Materials and methods) resulted in seven equally most parsimonious trees with a tree length of 447 steps, consistency index = 0.313 and retention index (RI) = 0.566 (electronic supplementary material, figure S6A). A strict consensus and a 50% majority-rule consensus tree were computed using these trees (figure 4; electronic supplementary material, figure S6B). In all trees, *Alienacanthus* is placed within Selenosteidae and, in the majority-rule consensus and four of the seven most parsimonious trees, it groups with other more derived Moroccan forms of the family, specifically with *Amazichthys*. In the majority consensus tree, this placement of *Alienacanthus* is supported by six characters: non-developed posterior margin of the rostral plate ((5) 1 => 0), anterior rostrum elongation ((6) 0 => 1), subequal external lengths of the preorbital and central plates ((15) 2 => 0), shallow postorbital embayment of the central plate ((20) 0 => 1), presence of a suborbital dermal lamina ((52) 0 —> 1) and posterior position of the junction between the postorbital, paranuchal and central plates relative to the anterior margin of the nuchal plate ((96) 0 => 1). *Alienacanthus* is placed at the base of a polytomy with *Amazichthys*, as a derived form within the Selenosteidae. In the three trees where *Alienacanthus* plots with *Amazichthys* as monophyletic, characters (5), (6) and (15) support the node. One tree plots *Alienacanthus* as sister group to the derived Moroccan selenosteids with characters (5), (6) and (96) supporting the node. In the other three trees, *Alienacanthus* plots with *Selenosteus*, a more basal form of the family (electronic supplementary material, figure S6). This affinity presents a node supported by the characters (6), (8), (13), (30), and (61). This uncertainty regarding the affinities of *Alienacanthus* might be owing to the incompleteness of the material, i.e. the lack of a preserved thoracic armour. It is possible that additional characters will contribute to the improvement of the family's phylogeny in the future as previous phylogenies have also shown some instability, demonstrated by variation within taxa placements in the family tree combined with an RI always above 0.5 [7,24,26].

## 4. Discussion

### 4.1. Extreme jaw elongation and modularity through time

**Figure 5.**
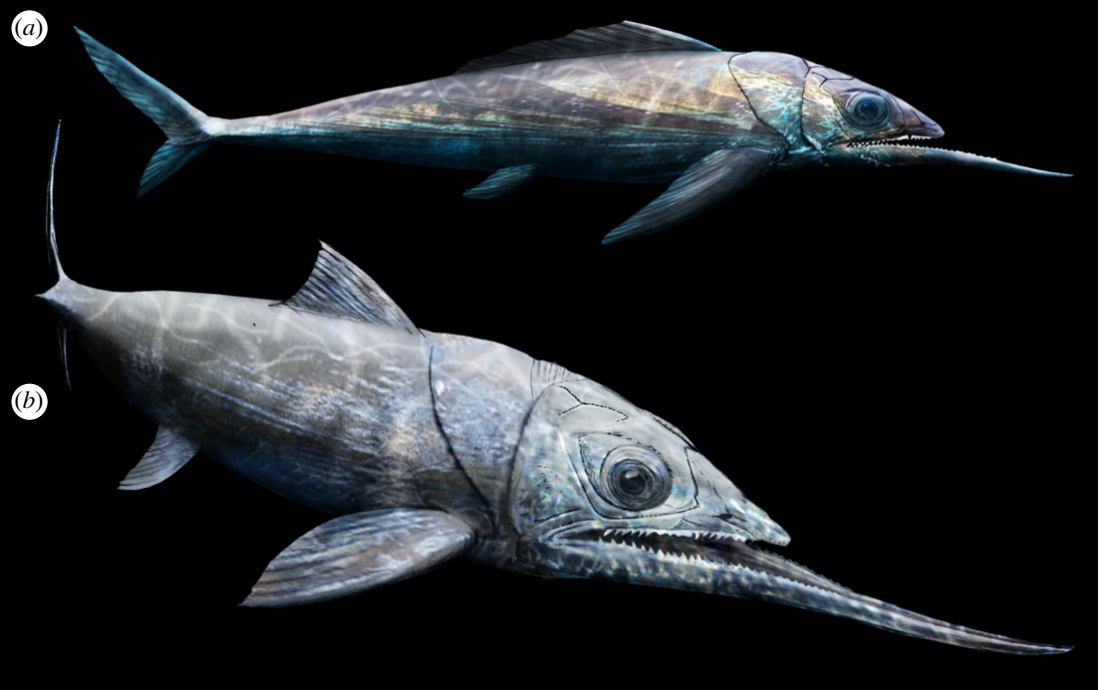
Live reconstructions of *Alienacanthus*. Based on the body morphology of extinct and modern fishes with elongated jaws (elongated, fusiform, bodies). Artwork by Beat Scheffold (Zürich) and Christian Klug.

The concept of modularity argues that, within a system, components can evolve independently from each other. Based on this concept, one would argue it would be difficult for many skull elements to be modular. For instance, selenosteids possess large orbits, a feature that involves changes in multiple skull bones [26]. The jaws, however, particularly the lower jaw, are elements that can change independently from the rest of the skull. The extreme lower jaw elongation in the Late Devonian placoderm *Alienacanthus* (figure 5) demonstrates the ancient evolvability of upper-lower jaw modularity and provides an example of the singular occupation of morphospace at the end of the **‘**age of fishes’ [12,37]. The conservation of molecular pathways and interactions involved in skeletal development across vertebrates [38] permits the reconstruction of developmental evolution in fossils [39,40]. The study of placoderms has been key in understanding the early evolution and evolvability of neural crest derived structures, including teeth and jaws [41,42]. Past research on jaw length regulation suggests that the anatomy of *Alienacanthus* most likely resulted from derived modifications in one or multiple pathways involving the transforming growth factor-beta, the bone morphogenetic proteins, the sonic hedgehog pathway members or the fibroblast growth factor [43–45]. In halfbeak fishes specifically, the calmodulin molecule paralogue calm1, known to play a generic role in bone cell proliferation, was identified as a potential regulator of jaw length, with its differential expression in upper and lower jaws [46]. This led to the development of the toothless protrusion in the lower jaw. Although *Alienacanthus* has no teeth on the anteriormost section of the IGs, a portion of the tooth-bearing component remains present past the occlusion with the upper gnathal elements. In any case, *Alienacanthus* shows an extreme dissociation in the length of upper and lower jaws, documented very few times across about 440 million years of jawed vertebrate evolution.

The extreme extent of the lower jaw elongation independent of the upper jaw is similar to that of three marine vertebrate taxa: the Carboniferous chondrichthyan *Ornithoprion* [47]; the ray-finned halfbeak fishes (Hemiramphidae) including their fossil record extending back to the Palaeogene [32]; and the Pliocene porpoise *Semirostrum* [48] (figure 4). Respectively, the lower jaws of these three taxa are 1.3, 1.6, and 1.2 times the length of the skull, less than the ratio of 2 for *Alienacanthus*. The two IGs of *Alienacanthus* were not fused in contrast to the lower jaws of the other taxa with extreme lower jaw elongation. The presence of a depression on the medial side of the IGs (figure 2*g*) suggest that there may have been some ligament or cartilage attached in the area to reinforce jaw connection. It is likely that the elements were in contact or near contact anterior to this attachment. In addition, the animals mentioned above date back to the Palaeogene (halfbeaks), Pliocene (*Semirostrum*) and Carboniferous (*Ornithoprion*). This makes *Alienacanthus* the earliest vertebrate displaying such elongation of the lower jaw.

### 4.2. Jaw morphology and anatomy

The unique jaw configuration of *Alienacanthus* must have resulted in species-specific behaviours including feeding, for which we can offer hypotheses based on the anatomical information derived from the several fossils reported here.

The unusual and extreme shape of the gnathal elements and their position in the articulated specimens suggest that the occlusion occurred only laterally without an anteriorly enlarged occlusion surface (electronic supplementary material, figure S5). This is a feature unique to *Alienacanthus* as arthrodiran placoderm jaws are normally rounded anteriorly (both ASG and IG), forming an occlusion laterally and anteriorly (i.e. U-shaped) (e.g. [26,35,49–51]). In *Alienacanthus*, the occlusion of the upper with the lower jaws continues anteriorly past the rostral plate and the ASGs do not meet. This is because the IGs are medial when occluding with the superognathals, thus preventing a contact between the ASGs anteriorly. Regarding the upper jaws' lateral articulation, the orientation of the dorsal process in the ASGs and PSGs is different to what has been documented so far in arthrodires. The dorsal lamina is in contact with regions of the ethmoid part of the neurocranium (for the ASG), the autopalatine (for the PSG), and a part of the palatoquadrate [30]. The dorsal processes of the upper gnathal plates in arthrodires, where present, are horizontal, forming an internal open near 90° angle with the occluding surface of the gnathal plates. In *Alienacanthus*, the processes are on the same axis as the shearing surface, making it unique to what was known so far in placoderms (e.g. [8,14,26,51–53]) (electronic supplementary material, figure S5C). This unique articulation also suggests that there was some flexibility in the movement of the ASGs medially.

As in other arthrodires like *Compagopiscis* [54] or *Plourdosteus* [55], there are two ossifications in the IG of *Alienacanthus*: the internal shaft and the tooth-bearing bone (figure 3). There are, however, key features that differ between the former and the latter. The last added tooth is the most posterior one in *Alienacanthus*, which implies tooth addition occurred posteriorly only. This is different from other derived forms like *Compagopiscis*, in which the dental ossification growth occurs in anterior, posterior and lingual directions [54,55]. Additionally, the margin between the tooth-bearing bone and the internal shaft is tight, i.e. the external border of outer layers forms only one dense layer, and the biting region does not surround the shaft ventrally and dorsally. By contrast, *Compagopiscis* and *Plourdosteus* show a distinct delimitation between the two ossifications’ outer compact layer at their contact margin and the biting division surrounds the internal shaft ventrally, laterally and dorsally. Moreover, in the thin section, the trabecular bone of *Alienacanthus* is spongier than that of *Compagopiscis* and *Plourdosteus*.

All gnathal elements bear posteriorly and slightly medially recurved teeth. This is a feature also seen in the enigmatic aspinothoracids *Diplognathus mirabilis* [56,57] and *Diplognathus lafargei* [57] from the Cleveland shale of Ohio. The jaws comprise long and slender IGs that form a fork-like symphysis at the tip, and possess posteriorly recurved teeth. Like *Alienacanthus*, *Diplognathus* is a rare taxon showing a unique jaw morphology with a large portion of the bone occupied by the occlusal region (over 50%). Other placoderms with recurved teeth include the selenosteid *Draconichthys elegans*, although these are recurved lingually only [26], and the dinichthyid *Hadrosteus rapax* with anteriorly recurved teeth [58,59].

### 4.3. *Alienacanthus* versus other arthrodire feeding strategies

The recurved teeth of the gnathal plates suggest that they functioned to capture live prey, such as fishes, and to direct it towards the oesophagus, as in many reptiles like snakes [60], pliosaurs [61] and choristoderans [62], or in fishes like the bowfin fish [63], northern snakehead or Atlantic salmon [63]. Another remarkable characteristic of the lower jaw is the continuation of the teeth beyond the occlusion. Several teeth lie anterior to this surface, *ca* 12 in the incomplete IG of PIMUZ A/I 5239. The presence of teeth anterior to the occlusal surface is seen in the ichthyosaurs *Eurhinosaurus* [64] and *Excalibosaurus* [65], as well as in two extant and one extinct chondrichthyan groups, the Pristiophoridae (sawsharks), the Pristidae (sawfish/carpenter sharks) and the Sclerorhynchoidei (rajiform ray). In these taxa, this feature lies in the upper jaw or rostrum. In the chondrichthyan groups, their rostra are equipped with sensory organs that allow detection and monitoring of movement near the animal through electroreception [66–68], although this is not limited to elongated jaws. Their teeth are used as a tool with the rostrum to strike the prey after detection [68]. Micro-teeth were reported and studied in three billfishes (*Kajikia audax*, *Istiophorus platypterus* and *Makaira nigricans*), for which they were also used to perform high speed dashes or precise strikes to attack prey of various sizes (large prey for *Makaira* and smaller prey from schools for *Kajikia* and *Istiophorus*) [69]. Electro-sensitivity was also suggested to be present in the lower jaw of *Semirostrum* [48] and, phylogenetically closer, in the elongated rostrum of the placoderm *Carolowilhelmina* [70]. Thin sections of *Alienacanthus* in the protruding section of the jaw do not indicate the presence of specialized nerve canals related to such function. This suggests that there was no increased sensitivity in its lower jaw. *Semirostrum* and halfbeaks bear a jaw that protrudes in a different way than in *Alienacanthus*; both have a normal length occlusal surface, i.e. the extension is edentulous, like a long chin. *Doryaspis* is a Devonian heterostracan (jawless vertebrate) with a long ventral pseudorostrum, comparable to the chin in gnathostomes [71–73]. Specifically, *Doryaspis nathorsti* [72,73] is equipped with small hook-shaped tubercles on the pseudorostrum's lateral margins resembling teeth. *Alienacanthus* has teeth that continue past the occlusal surface in addition to jaw elongation. We suggest that the anteriormost teeth were part of the occlusion at an earlier ontogenetic stage but, with growth of the IGs, these teeth moved out of the occlusal surface. We propose either a sudden growth of the lower jaw as in the halfbeak [74] or a retarded growth of the upper jaws, as seen in the mandible of the swordfish [75]. It is possible that the animal used its extremely long jaw to strike and confuse prey like other large animals with jaw elongation such as *Eurhinosaurus* [64] or *Xiphias* [76], and that these anterior teeth could be used to harm soft-bodied prey.

Arthrodires display a range of jaw morphologies, which reflect a variety of functions and adaptations, particularly in the Late Devonian [4,11,12,77]. Some taxa continued favouring speed and strength, like *Dunkleosteus* [78]. Others developed different types of feeding, such as the potentially filter feeding *Titanichthys* [13]. More jaw disparity is visible in the Australian Gogo Formation with the durophagous *Bruntonichthys*, *Bullerichthys* and *Kendrickichthys* [14] and the supposed grazer *Incisoscutum*, with jaws adapted to catching soft resilient prey in the reefs or the water column [79]. Like in *Incisoscutum*, *Alienacanthus* jaws do not appear to be adapted for high strength or speed, as fast and strong jaws were typically made of blades and reduced teeth [11,79]. Although further information concerning the hyoid and hypobranchial apparatus could bring additional insights into potential jaw function, it is likely that such jaws would develop as an adaptation to a feeding or hunting method. This coincides well with the presence of recurved teeth on the gnathal plates.

**Figure 6.**
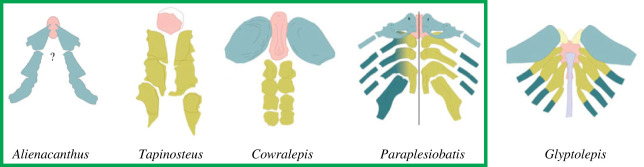
Pharyngeal gill arches of placoderms. Reconstructed gill arches of placoderms (in green box) and lobe-finned fish *Glyptolepis* from [80]. *Alienacanthus* only has the first arch preserved and thus the basihyal is subjective and the other arches unknown. Colours: pink, basihyal; blue, first arch; green, basibranchials; dark blue, ceratobranchials. Image not to scale.

The first gill arch of *Alienacanthus* can be compared to that of other placoderms (figure 6). The sub-triangular shape of the ceratohyal is most similar to that of the pseudopetalichthyid *Paraplesiobatis*, although the latter may be more complex and composed of both the ceratohyal and first branchial arch. The lateral segment is larger and thicker than that of other early vertebrates, including that of *Paraplesiobatis*, and the chondrichthyans *Acanthodes*, *Ozarcus* and *Debeerius* [80]. This structure may have provided strength and support for the elongated lower jaws.

### 4.4. Palaeogeographical context

During the Famennian, both the Holy Cross Mountains and the Anti-Atlas were part of the Rheic Ocean, connected to the Palaeotethys [81]. McKerrow *et al*. [82] argued that the Rheic Ocean was small enough to allow migration of faunas between Laurussia (Laurasia and Baltica) and Gondwana. This is supported by the occurrence of similar taxa in both parts of the Palaeotethys [26,83–86], indicating a close palaeogeographical relationship as suggested by Rücklin [26,87] for the Frasnian placoderm assemblages. The presence of *Alienacanthus* in both Gondwana and Baltica (figure 1*a*) concurs with the absence of a palaeogeographical barrier between Gondwana and Laurussia at that time. Together with the streamlined shape of the skull, this suggests that *Alienacanthus* was a pelagic swimmer, that inhabited much of the Palaeotethys.

## 5. Conclusion

The study of the anatomy, phylogenetic relationships and ecomorphological features of *Alienacanthus* is an example of how exceptionally preserved fossils can provide direct evidence on developmental evolution and morphospace occupation that would remain unsuspected and unknowable based on studies of extant species alone.

## Data Availability

The datasets supporting this article have been uploaded as part of the electronic supplementary material [88].
